# Supplementary material for: Kupffer cells determine intrahepatic traffic of PEGylated liposomal doxorubicin
Source: Nat Commun. 2024 Jul 20;15:6136. doi: 10.1038/s41467-024-50568-7 (PMC11271521; doi:10.1038/s41467-024-50568-7)
Supplement: Supplementary file 1 — Supplementary Information [file 41467_2024_50568_MOESM1_ESM.pdf]

Kupffer cells determine intrahepatic traffic of PEGylated liposomal doxorubicin

Kuan Jiang[1,2,*], Kaisong Tian[2], Yifei Yu[2], Ercan Wu[2], Min Yang[2], Feng Pan[3], Jun Qian[3], Changyou Zhan[2,3,*]

[1]Eye Institute and Department of Ophthalmology, Eye & ENT Hospital, Fudan University, Shanghai 200030, P.R. China

[2]Department of Pharmacology, School of Basic Medical Sciences & Department of Pharmacy, Shanghai Pudong Hospital & State Key Laboratory of Molecular Engineering of Polymers, Fudan University, Shanghai 200032, P.R. China

[3]School of Pharmacy, Fudan University & Key Laboratory of Smart Drug Delivery (Fudan University), Ministry of Education, Shanghai, 201203, P.R. China.

*To whom correspondence should be addressed. Tel: 86-21-54237379. E-mail: cyzhan@fudan.edu.cn (CZ) or jiangkuan@fudan.edu.cn (KJ)

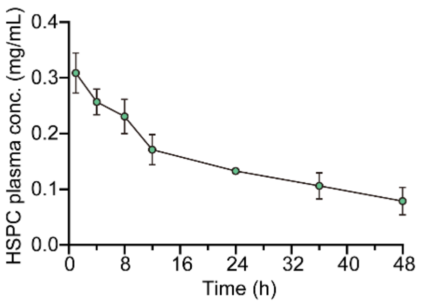

Supplementary Figure 1. Pharmacokinetic profile of 5-carboxyfluorescein (FAM) labeled PEGylated liposomes (FAM-sLip) in C57BL/6J mice. The FAM-sLip was intravenously injected at a dose of 50 mg/kg HSPC. Data are means ± SDs (n = 3 mice).

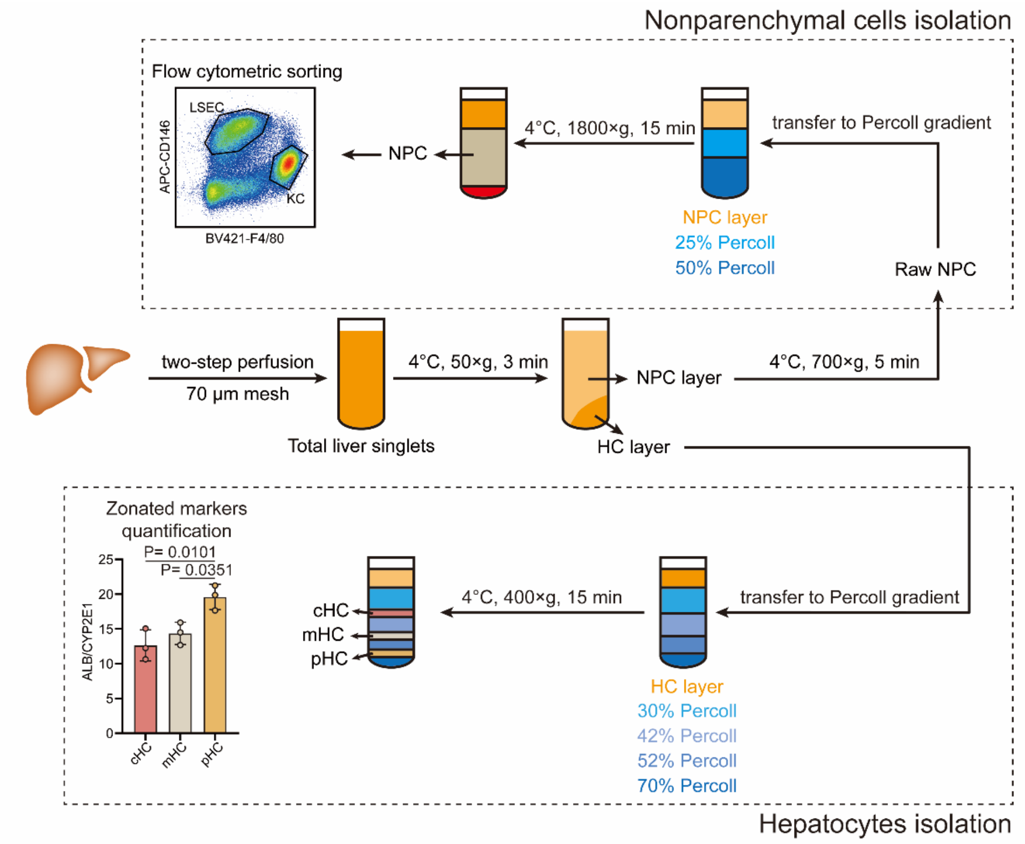

Supplementary Figure 2. A facile proceedure for liver cells isolation. Briefly, after a two-step perfusion digestion, liver was cut into pieces and filter through a 70 μm meshed strainer to obtain the total liver cells singlets. Then hepatocytes (HC) and nonparenchymal cells (NPC) were separated through different centrifugal forces (50×g for HC while 700×g for NPC), and transferred to Percoll gradient for further isolation. HC along the lobule porto-central axis was seperated through a 30%/42%/52%/70% Percoll gradient. The statistical significance was analyzed by one-way ANOVA multiple comparisons corrected by Tukey's test. Data are means ± SDs (n = 3 mice). cHC, pericentral hepatocytes; mHC, mid-lobule hepatocytes; pHC, periportal hepatocytes. The intrahepatic Kupffer cells (KC), liver sinusoidal endothelial cells (LSEC), B lymphocytes and T lymphocytes were obtained conbined 25%/50% Percoll gradient and flow cytometric sorting as in "Liver cells isolation" part in Methods.

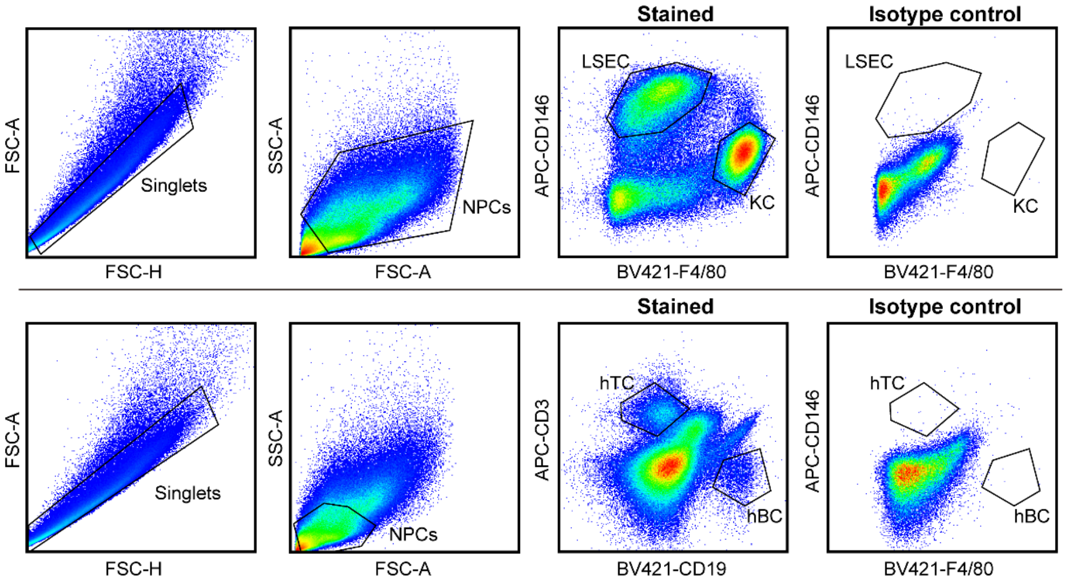

Supplementary Figure 3. Gating strategy of fluorescence activated cell sorting for intrahepatic Kupffer cells (KC), liver sinusoidal endothelial cells (LSEC), B lymphocytes (hBC) and T lymphocytes (hTC). The parameters of FSC-A and FSC-H were used for selection of singlets. FSC-A and SSC-A were used for size selection of special nonparenchymal cells (NPC). The cell types were defined as KC (F4/80$^+$), LSEC (CD146$^+$), hBC (CD19$^+$) and hTC (CD3$^+$).

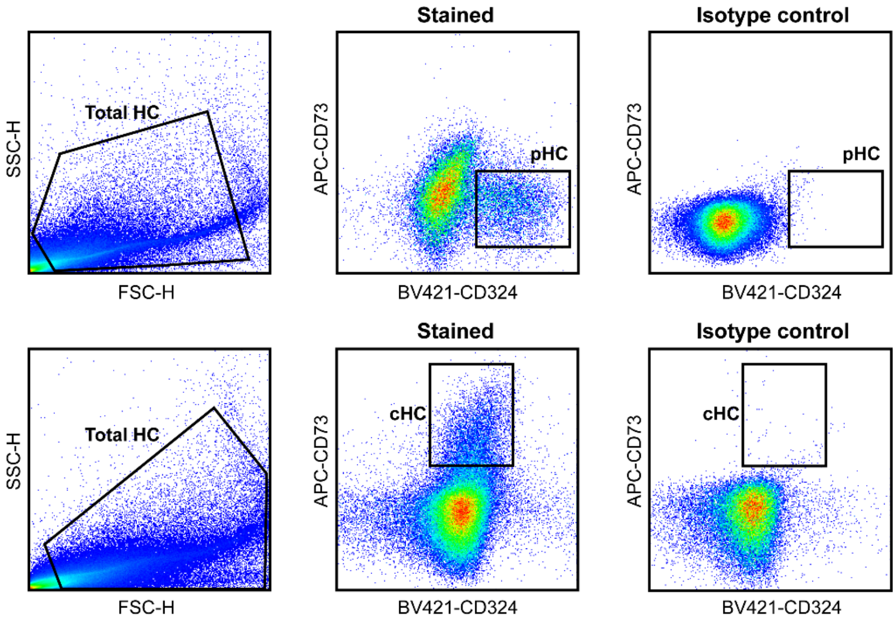

Supplementary Figure 4. Gating strategy of fluorescence activated cell sorting for periportal hepatocytes (pHC) and pericentral hepatocytes (cHC). The parameters of FSC-H and SSC-H were used for size selection of total HC. The cell types were defined as pHC ($CD324^+$) and cHC ($CD73^+$).

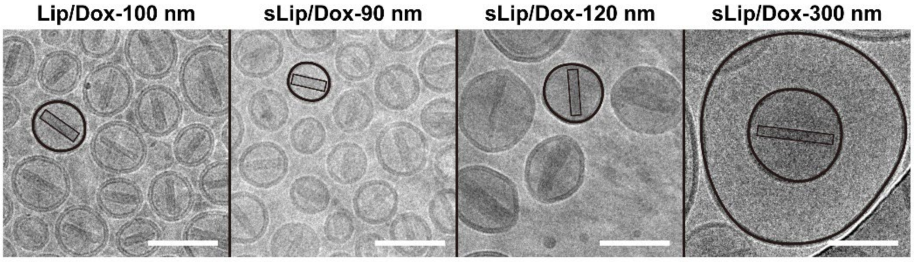

Supplementary Figure 5. Cryo-TEM pictures of non-PEGylated liposomal doxorubicin (Lip/Dox) and PEGylated liposomal doxorubicin (sLip/Dox) with different particle sizes. The lipid membrane was labeled by the circle while doxorubicin nanocrystal was labeled by the rectangle. Scale bar, 100 nm.

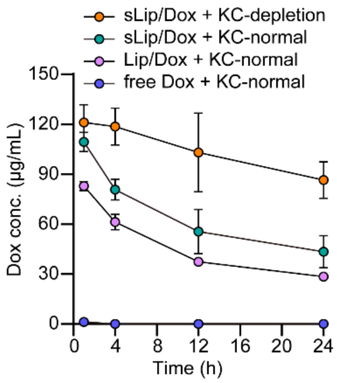

Supplementary Figure 6. Pharmacokinetic profiles of sLip/Dox, Lip/Dox and free Dox in C57BL/6J mice. The mice were intravenously injected with different preparations at a dose of 5 mg/kg Dox. Data are means ± SDs (n = 3 mice).

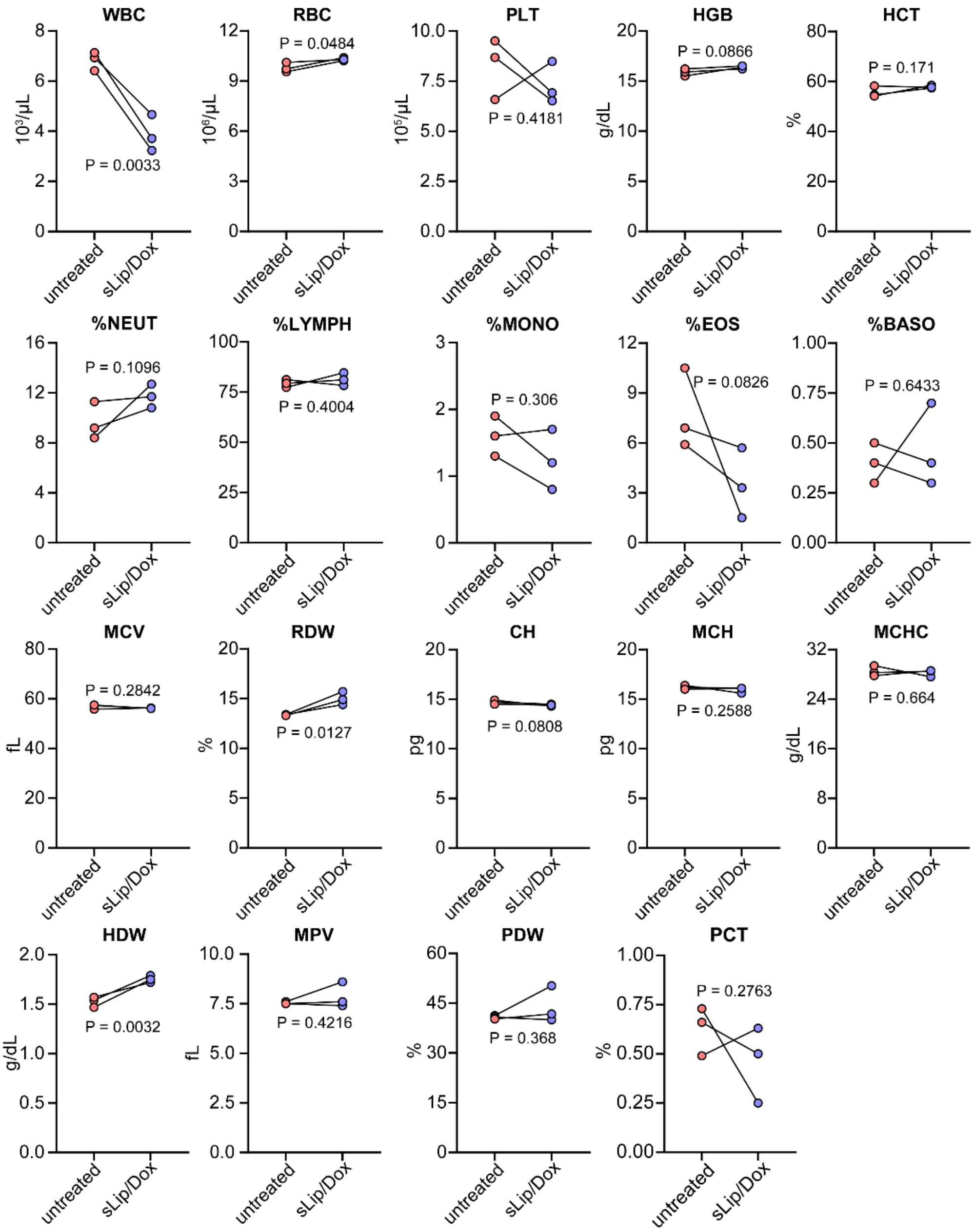

Supplementary Figure 7. Routine blood tests of the peripheral blood from untreated mice and mice treated by sLip/Dox. The treated mice were intravenously injected with sLip/Dox at a dose of 5 mg/kg doxorubicin, and the whole blood was gathered at 24 h after injection in EDTA-containing tube at room temperature before test. The statistical significance was analyzed by two-tailed unpaired t-test. Data are means ± SDs (n = 3 mice). WBC, white blood cell; RBC, red blood cell; PLT, platelet; HGB, hemoglobin; HCT, hematocrit; NEUT, neutrophil; LYMPH, lymphocyte; MONO, monocyte; EOS, eosinophilic; BASO, basophil; MCV, mean corpuscular volume; RDW, red cell distribution width; CH, corpuscular hemoglobin; MCH, mean corpuscular hemoglobin; MCHC, mean corpuscular hemoglobin concentration; HDW, hemoglobin distribution width; MPV, mean platelet volume; PDW, platelet volume distribution width; PCT, platelet crit.

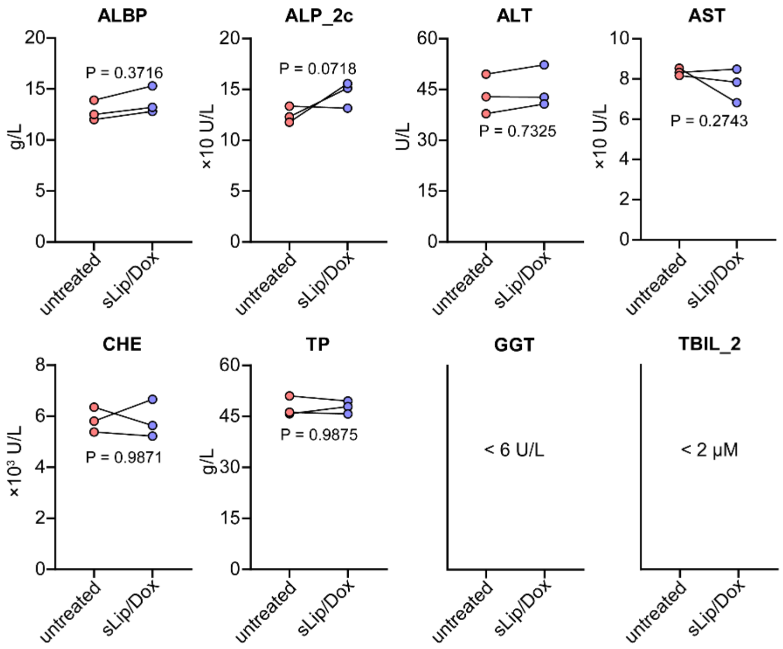

Supplementary Figure 8. Liver function evaluation reflecting by serum biochemical analysis. The treated mice were intravenously injected with sLip/Dox at a dose of 5 mg/kg doxorubicin, and the whole blood was gathered at 24 h after injection in tube at room temperature before test, further to keep at room temperature for 1 h to obtain the serum. The statistical significance was analyzed by two-tailed unpaired t-test. Data are means ± SDs (n = 3 mice). ALB, albumin; ALP_2c, alkaline phosphatase 2c; ALT, alanine aminotransferase; AST, aspertate aminotransferase; CHE, cholinesterase; TP, total protein; GGT, glutamyltransferase; TBIL_2, total bilirubin_2.

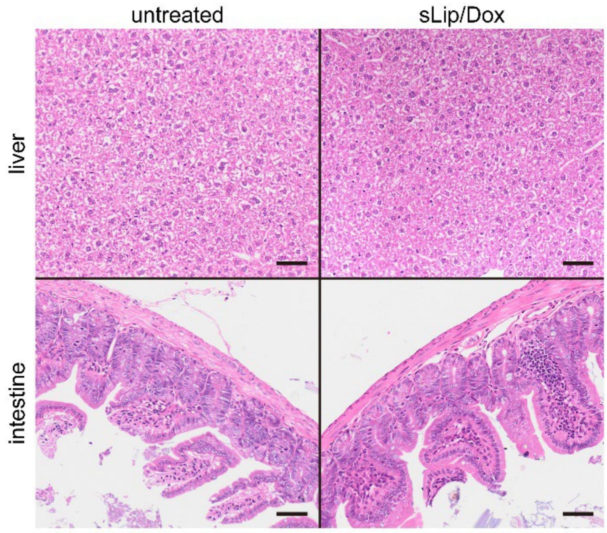

Supplementary Figure 9. Hematoxylin-eosin staining sections of liver or intestine. Mice in sLip/Dox treated group were intravenously injected with sLip/Dox at a dose of 5 mg/kg doxorubicin. The tissues were gathered 24 h after injection. Scale bar, 50 μm.

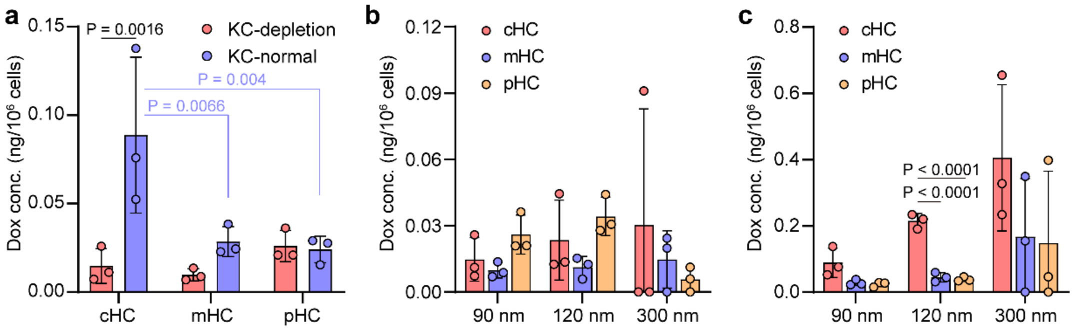

Supplementary Figure 10. Distribution of sLip/Dox in zonated HC at different conditions. (**a**) Distribution of sLip/Dox (90 nm) in zonated HC at 12 h post injection with KC depletion or not. (**b**) Distribution of sLip/Dox (90 nm, 120 nm or 300 nm) in zonated HC at 12 h post injection with KC depletion. (**c**) Distribution of sLip/Dox (90 nm, 120 nm or 300 nm) in zonated HC at 12 h post injection in KC-normal condition. The sLip/Dox was intravenously injected at a dose of 5 mg/kg Dox. The statistical significance was analyzed by 2-way ANOVA multiple comparisons corrected by Tukey's or Sidak's test for **a**, and by one-way ANOVA multiple comparisons corrected by Tukey's test for **b-c**. P values are provided when there are statistical significances ($P < 0.05$). Data are means ± SDs (n = 3 mice).

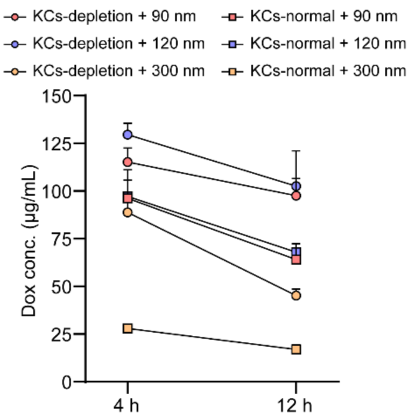

Supplementary Figure 11. Pharmacokinetic profiles of sLip/Dox in mice. The groups were set as in **Figure 3**. The C57BL/6J mice were intravenously injected with sLip/Dox at a dose of 5 mg/kg Dox. Data are means ± SDs (n = 3 mice).

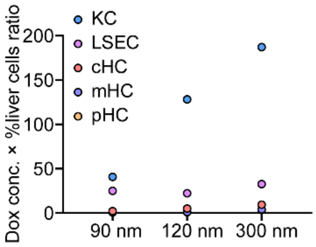

Supplementary Figure 12. Rough calculation of total Dox distribution in major liver cells at 12 h after injection. Data were calculated as in Figure 3h.

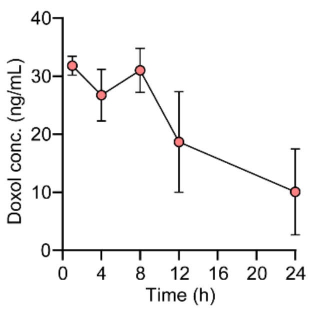

Supplementary Figure 13. Pharmacokinetic profiles of doxorubicinol (Doxol) due to metabolism of Dox encapsulated in liposomes. The C57BL/6J mice were intravenously injected with sLip/Dox at a dose of 5 mg/kg Dox. Data are means ± SDs (n = 3 mice).

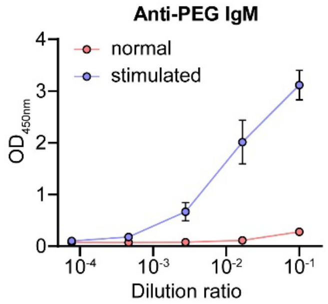

Supplementary Figure 14. Anti-PEG IgM level of C57BL/6J mice pretreated with sLip at a dose of 5 mg/kg HSPC. The serum from pretreated mice was extracted 5 d after single dose of intravenous injected sLip, and the anti-PEG IgM level was detected by an ELISA method. Data are means ± SDs (n = 5 for stimulated mice while n = 2 for normal mice).

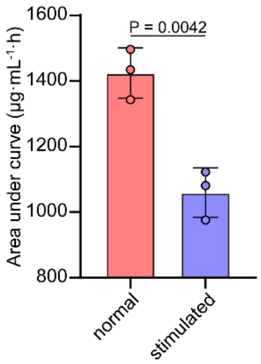

Supplementary Figure 15. Area under curve of plasma Dox concentration. The normal mice were intravenously injected with sLip/Dox at a dose of 5 mg/kg Dox, while the stimulated mice were treated at the same dose 5 days after pre-stimulated. The statistical significance was analyzed by two-tailed unpaired t test. Data are means ± SDs (n = 3 mice at each timepoint).

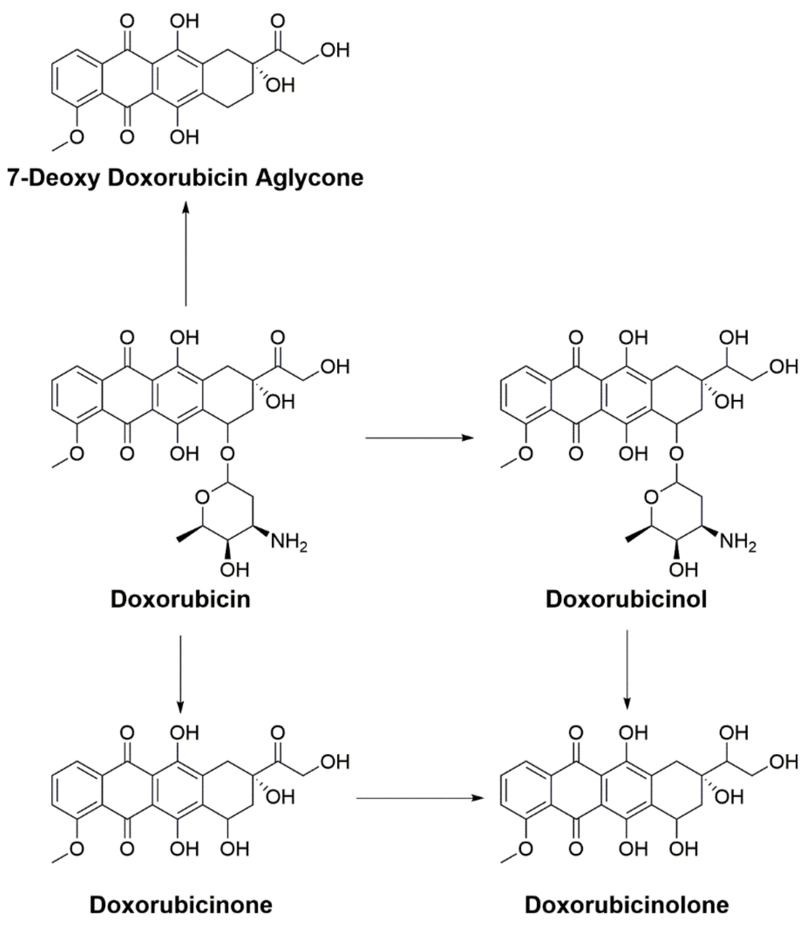

Supplementary Figure 16. Molecular structures of Dox and the main metabolites.

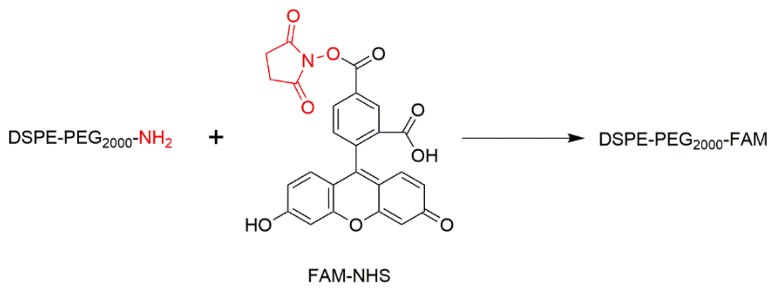

Supplementary Figure 17. Synthesis of FAM labeled DSPE-PEG$_{2000}$ (DSPE-PEG$_{2000}$-FAM). DSPE-PEG$_{2000}$-NH$_2$ and FAM-NHS were dissolved in DMSO containing 1% (v/v) trimethylamine, following stirring at room temperature overnight. DSPE-PEG$_{2000}$-FAM was obtained after dialysis and lyophilization.

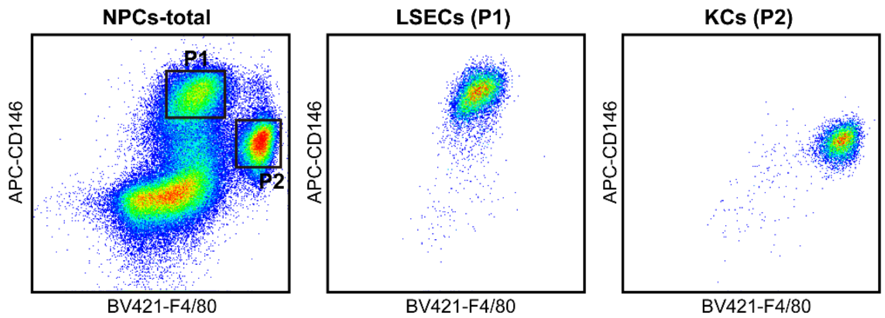

Supplementary Figure 18. Fluorescence activated cell sorting gating strategy for LSECs and KCs from total NPCs. The KCs were defined as F4/80$^+$ while LSECs were defined as CD146$^+$.

Supplementary Table 1. Particle sizes, polydispersity index (PDI) and surface zeta potential (ZP) of FAM-sLip and sLip/Dox.

	Size (d. nm)	PDI	ZP (mV)	LC/%	EE/%
FAM-sLip	83.7 ± 0.9	0.037 ± 0.031	-19.4 ± 0.8	-	-
sLip/Dox (90 nm)[a]	87.5 ± 1.0	0.108 ± 0.036	-22.5 ± 1.0	14.5 ± 0.5	98.6 ± 0.5
sLip/Dox (120 nm)	120.7 ± 1.0	0.113 ± 0.012	-16.9 ± 1.6	12.8 ± 0.6	96.0 ± 0.3
sLip/Dox (300 nm)	293.6 ± 11.2	0.132 ± 0.035	-18.7 ± 1.1	7.30 ± 0.2	95.5 ± 0.6
Lip/Dox	102.1 ± 2.4	0.049 ± 0.034	-8.16 ± 0.7	12.4 ± 0.7	96.7 ± 0.5

a. sLip/Dox used in this work was with a particle size of 90 nm except particularly statement.
PDI, polydispersity index; ZP, surface zeta potential; LC, drug loading capacity, calculated by the ratio of doxorubicin concentration to HSPC concentration; EE, encapsulation efficiency, calculated as the ratio of free doxorubicin concentration to total doxorubicin in sLip/Dox.

Supplementary Table 2. Concentrations of albumin (ALB) and cytochrome P450 (CYP2E1) in mice hepatocytes from different lobule zones.

	CV-HCs	Mid-HCs	PN-HCs
ALB (μg/mL)	14.2 ± 4.07	12.0 ± 1.49	19.9 ± 7.16
CYP2E1 (ng/mL)	1.11 ± 0.19	0.85 ± 0.17	1.05 ± 0.46
ALB/CYP2E1	12.6 ± 1.82	14.4 ± 1.32	19.6 ± 1.48

The concentration of special proteins was detected refered to instruction book of the ELISA kit (Shanghai Enzyme-linked Biotechnology, ml037889 for ALB, ml037417 for CYP2E1).
The values of ALB/CYP2E1 were calculated as the ratios of ALB to CYP2E1 in the same hepatocytes sample from the one mouse, without consideration in unit conversion. CV-HCs, hepatocytes near the central vein; Mid-HCs, hepatocytes in middle lobule zone; PN-HCs, hepatocytes near the portal node.
