# Supplementary material for: Kupffer cells determine intrahepatic traffic of PEGylated liposomal doxorubicin
Source: Nat Commun. 2024 Jul 20;15:6136. doi: 10.1038/s41467-024-50568-7 (PMC11271521; doi:10.1038/s41467-024-50568-7)
Supplement: Supplementary file 3 — Reporting Summary [file 41467_2024_50568_MOESM3_ESM.pdf]

nature portfolio

Corresponding author(s): Kuan Jiang, Changyou Zhan

Last updated by author(s): Jul 3, 2024

Reporting Summary

Nature Portfolio wishes to improve the reproducibility of the work that we publish. This form provides structure for consistency and transparency in reporting. For further information on Nature Portfolio policies, see our Editorial Policies and the Editorial Policy Checklist.

Statistics

For all statistical analyses, confirm that the following items are present in the figure legend, table legend, main text, or Methods section.

n/a	Confirmed	
☐	☒	The exact sample size (*n*) for each experimental group/condition, given as a discrete number and unit of measurement
☐	☒	A statement on whether measurements were taken from distinct samples or whether the same sample was measured repeatedly
☐	☒	The statistical test(s) used AND whether they are one- or two-sided<br>*Only common tests should be described solely by name; describe more complex techniques in the Methods section.*
☐	☒	A description of all covariates tested
☐	☒	A description of any assumptions or corrections, such as tests of normality and adjustment for multiple comparisons
☐	☒	A full description of the statistical parameters including central tendency (e.g. means) or other basic estimates (e.g. regression coefficient) AND variation (e.g. standard deviation) or associated estimates of uncertainty (e.g. confidence intervals)
☐	☒	For null hypothesis testing, the test statistic (e.g. *F*, *t*, *r*) with confidence intervals, effect sizes, degrees of freedom and *P* value noted<br>*Give P values as exact values whenever suitable.*
☒	☐	For Bayesian analysis, information on the choice of priors and Markov chain Monte Carlo settings
☒	☐	For hierarchical and complex designs, identification of the appropriate level for tests and full reporting of outcomes
☒	☐	Estimates of effect sizes (e.g. Cohen's *d*, Pearson's *r*), indicating how they were calculated

Our web collection on statistics for biologists contains articles on many of the points above.

Software and code

Policy information about availability of computer code

Data collection	N/A
Data analysis	GraphPad Prism 8.0.

For manuscripts utilizing custom algorithms or software that are central to the research but not yet described in published literature, software must be made available to editors and reviewers. We strongly encourage code deposition in a community repository (e.g. GitHub). See the Nature Portfolio guidelines for submitting code & software for further information.

Data

Policy information about availability of data

All manuscripts must include a data availability statement. This statement should provide the following information, where applicable:

- Accession codes, unique identifiers, or web links for publicly available datasets
- A description of any restrictions on data availability
- For clinical datasets or third party data, please ensure that the statement adheres to our policy

Data supporting the findings of this study are provided in the main manuscript/Supplementary Information/Source Data file. Source data are provided with this paper.

Research involving human participants, their data, or biological material

Policy information about studies with human participants or human data. See also policy information about sex, gender (identity/presentation), and sexual orientation and race, ethnicity and racism.

Reporting on sex and gender	No related information in this study.
Reporting on race, ethnicity, or other socially relevant groupings	No related information in this study.
Population characteristics	No related information in this study.
Recruitment	No related information in this study.
Ethics oversight	No related information in this study.

Note that full information on the approval of the study protocol must also be provided in the manuscript.

Field-specific reporting

Please select the one below that is the best fit for your research. If you are not sure, read the appropriate sections before making your selection.

☒ Life sciences ☐ Behavioural & social sciences ☐ Ecological, evolutionary & environmental sciences

For a reference copy of the document with all sections, see nature.com/documents/nr-reporting-summary-flat.pdf

Life sciences study design

All studies must disclose on these points even when the disclosure is negative.

Sample size	The numbers of animals in every group were no less than 3 different animals (except 2 for normal mice in anti-PEG IgM detection) and tested independently to enable the further statistical analysis.
Data exclusions	No data were excluded from the analyses.
Replication	All replication were independent.
Randomization	Samples were randomly allocated into groups.
Blinding	The investigators were blinded to group allocation during data collection and analysis.

Reporting for specific materials, systems and methods

We require information from authors about some types of materials, experimental systems and methods used in many studies. Here, indicate whether each material, system or method listed is relevant to your study. If you are not sure if a list item applies to your research, read the appropriate section before selecting a response.

Materials & experimental systems

n/a	Involved in the study	
☐	☒	Antibodies
☒	☐	Eukaryotic cell lines
☒	☐	Palaeontology and archaeology
☐	☒	Animals and other organisms
☒	☐	Clinical data
☒	☐	Dual use research of concern
☒	☐	Plants

Methods

n/a	Involved in the study	
☒	☐	ChIP-seq
☐	☒	Flow cytometry
☒	☐	MRI-based neuroimaging

Antibodies

Antibodies used	HRP-labeled goat anti-mouse IgM mu chain, Abcam, Cat#AB97230, Lot#1028601-18; BV421 anti-mouse F4/80, Biolegend, Cat#123137, BM8, Lot#B422926; APC anti-mouse CD146, Biolegend, Cat#134712, ME-9F1, Lot#B396861; BV421 anti-mouse CD19, Biolegend, Cat#115538, 6D5, B399079; APC anti-mouse CD3, Biolegend, Cat#100236, 17A2, Lot#B413435; BV421 anti-mouse CD324,

Biolegend, Cat#147319, DECMA-1, Lot#B324426; APC anti-mouse CD73, Biolegend, Cat#127210, TY/11.8, Lot#B291453; BV421 anti-rat IgG2a, Biolegend, Cat#400535, RTK2758, Lot#B340002; APC anti-rat IgG2a, Biolegend, Cat#400511, RTK2758, Lot#B336072; APC anti-rat IgG2b, Biolegend, Cat#400611, RTK4530, Lot#B314628; BV421 anti-rat IgG1, Biolegend, Cat#400429, RTK2071, Lot# B343563; APC anti-rat IgG1, Biolegend, Cat#400411, RTK2071, Lot#B337839; FcR Blocking Reagent, Miltenyi, Cat#130-092-575, Lot#5240407396.

Validation

HRP-labeled goat anti-mouse IgM mu chain, https://www.abcam.cn/products/secondary-antibodies/goat-mouse-igm-mu-chain-hrp-ab97230.html; BV421 anti-mouse F4/80, https://www.biolegend.com/en-us/products/brilliant-violet-421-anti-mouse-f4-80-antibody-7199; APC anti-mouse CD146, https://www.biolegend.com/en-us/products/apc-anti-mouse-cd146-antibody-9289; BV421 anti-mouse CD19, https://www.biolegend.com/en-us/products/brilliant-violet-421-anti-mouse-cd19-antibody-7160; APC anti-mouse CD3, https://www.biolegend.com/en-us/products/apc-anti-mouse-cd3-antibody-8055; BV421 anti-mouse CD324, https://www.biolegend.com/en-us/products/brilliant-violet-421-anti-mouse-human-cd324-e-cadherin-antibody-16415; https://www.biolegend.com/en-us/products/apc-anti-mouse-cd73-antibody-7893; BV421 anti-rat IgG2a, https://www.biolegend.com/en-us/products/brilliant-violet-421-rat-igg2a-kappa-isotype-ctrl-7135; APC anti-rat IgG2a, https://www.biolegend.com/en-us/products/apc-rat-igg2a-kappa-isotype-ctrl-1838; APC anti-rat IgG2b, https://www.biolegend.com/en-us/products/apc-rat-igg2b-kappa-isotype-ctrl-1851; BV421 anti-rat IgG1, https://www.biolegend.com/en-us/products/brilliant-violet-421-rat-igg1-kappa-isotype-ctrl-7134; APC anti-rat IgG1, https://www.biolegend.com/en-us/products/apc-rat-igg1-kappa-isotype-ctrl-1826; FcR Blocking Reagent, https://www.miltenyibiotec.com/CN-en/products/fcr-blocking-reagent-mouse.html#130-092-575.

Animals and other research organisms

Policy information about studies involving animals; ARRIVE guidelines recommended for reporting animal research, and Sex and Gender in Research

Laboratory animals

Healthy male C57BL/6J mice (7-8 weeks) were obtained from the Experimental Animal Center of Fudan University, and maintained at 22 ± 2 °C and 50% ± 5% humidity on a 12 h light–dark cycle with access to standard rodent chow and water ad libitum. The other two animal models were obtained via intravenous injection of clodronate liposomes or PEGylated liposomes on those mice.

Wild animals

No wild animals involved in this study.

Reporting on sex

The findings in this study apply to only male mice considering male mice were usually with a more stable physiological status than the female counterpart.

Field-collected samples

This study did not involve samples collected from the field.

Ethics oversight

Animals used in this study were treated according to protocols that were approved by the Ethical Committee of Fudan University

Note that full information on the approval of the study protocol must also be provided in the manuscript.

Plants

Seed stocks

No related information in this study.

Novel plant genotypes

No related information in this study.

Authentication

No related information in this study.

Flow Cytometry

Plots

Confirm that:

☒ The axis labels state the marker and fluorochrome used (e.g. CD4-FITC).

☒ The axis scales are clearly visible. Include numbers along axes only for bottom left plot of group (a 'group' is an analysis of identical markers).

☒ All plots are contour plots with outliers or pseudocolor plots.

☒ A numerical value for number of cells or percentage (with statistics) is provided.

Methodology

Sample preparation

Cell samples were prepared as in Liver cells isolation part in Methods.

Instrument	Aglient Novocyte.
Software	Flowjo 10.0.0.
Cell population abundance	>5000 cells.
Gating strategy	Boundary was set when the "negative" (unstained cells) was <0.1%.

☒ Tick this box to confirm that a figure exemplifying the gating strategy is provided in the Supplementary Information.
